# Molecular characterization of infectious bronchitis viruses isolated from broiler flocks in Bushehr province, Iran: 2014 - 2015

**Published:** 2017-09-15

**Authors:** Yousef Saadat, Mohammad Hassan Bozorgmehri Fard, Saied Charkhkar, Hossein Hosseini, Nariman Shaikhi, Bijan Akbarpour

**Affiliations:** 1 Department of Poultry and obstetrics Sciences and Research Branch, Islamic Azad University, Tehran, Iran;; 2 Department of Clinical Sciences, Faculty of Veterinary Medicine, Karaj Branch, Islamic Azad University, Karaj, Iran;; 3 Department of Basic Sciences, Faculty of Veterinary Medicine, Kazerun Branch, Islamic Azad University, Kazerun, Iran.

**Keywords:** Infectious bronchitis virus, Massachusetts, RT-PCR, Variant 2

## Abstract

The aim of this study was to provide information on the molecular characteristic and the phylogenic relationship of infectious bronchitis viruses (IBV) strains in Bushehr province in comparison to other strains reported in the Middle East. Samples were collected from broiler flocks in Bushehr province during 2014 - 2015. These flocks had respiratory problems such as gasping, sneezing and bronchial rales. A number of 135 tracheal swabs were taken from fifteen flocks (nine swabs per flock). Each three swabs collected from each flock were pooled in one tube (finally, we had three tubes for each flock). The samples were subjected to reverse transcription polymerase chain reaction (RT-PCR). The PCR products of positive samples were analyzed by sequencing of a (392 bp) segment of the spike gene and the related results were compared with the other IBV sequences in GenBank database. Samples from twelve farms (80.0%) were found to be positive. The viruses from seven farms (46.6%) were identified as field viruses closely related to variant 2. The viruses from three farms (20.0%) were characterized as Mass type and were related to vaccine strains. Two different IB viruses (variant 2 and Mass) were detected in samples from two farms (13.3%). The variant 2 genotype detected in Bushehr had high similarity to variant 2 reported from the Middle East. These variants displayed homologies ranging from 72.9% to 76.5%, and 78.8% to 80.0% with H120 and 4/91, respectively. It is necessary to design vaccination program of poultry farms using IBV strains circulating in the region.

## Introduction

Infectious bronchitis (IB) is an acute and highly contagious respiratory disease of chickens characterized by respiratory signs, and in young chickens by severe respiratory distress and a decrease in egg production in layers.^1^ The chicken was considered the only natural host of infectious bronchitis virus (IBV) but recently pheasants has been introduced as the other natural host for IBV.^2^ The disease is transmitted by the respiratory route, direct contact and indirectly through mechanical spread.^3^ The virus belongs to *Coronaviridae*, Order *Nidovirales*. The IBV and other avian *coronaviruses* of turkeys and pheasants are classified as group 3 *coronaviruses*.^4^ Its genome consists of about 27 kb and codes for four structural proteins: the spike (S) glycoprotein, the membrane (M) glycoprotein, the nucleocapsid (N) phosphoprotein, and the envelope (E) protein.^5^^,^^6^ The spike glycoprotein (S) is anchored in the viral envelope and is post-translationally cleaved into two proteins S1 and S2.^7^ The S protein is very diverse in terms of both nucleotide sequence and deduced primary protein structure, especially in the upstream part of S1.^8^ Three hypervariable regions (HVRs) have been identified in the S1 subunit.^9^^-^^11^ The S1 subunit induces neutralizing, serotype-specific, and haemagglutination-inhibiting antibodies.^12^^-^^17^ Amino acid changes in the spike (S) glycoprotein lead to the generation of genetic variants.^18^^,^^19^ The high frequency of new IBV variants is a distinguished characteristic of this virus among other *coronaviruses*.^20^ Many IBV serotypes have been described probably due to the frequent point mutations that occur in RNA viruses and also recombination events. Therefore, the characterization of virus isolates which exists in the field is very important.^21^ More than 50 serotypes of IBV have been identified and new variants continued to emerge despite the use of live attenuated and killed IBV vaccines.^22^^-^^24^

The usage of live attenuated vaccines is the most important preventive measure of the disease, but anti-genically different serotypes and newly emerged variants from field chicken flocks sometimes cause vaccine breaks.^18^^,^^19^ The IBV Massachusetts (Mass) type was first detected in Iran by Aghakhan *et al*.^25^ In 1998, a virus similar to the European 793/B type was isolated in Iran (Iran/793B/19/08).^26^ In recent years, new variants of IBV have been reported from different part of the country.^27^^-^^29^ The aim of this study was to provide information on the molecular characteristic and the phylogenetic relationship of prevalent IBV genotypes circulating in chicken flocks in Bushehr province, Iran.

## Materials and Methods

**Sampling.** Samples were collected from broiler flocks in different regions of Bushehr province as mentioned in Table 1 during 2014-2015. These flocks showed respiratory problems such as gasping, sneezing and bronchial rales. A number of 135 tracheal swabs were taken from fifteen flocks (nine swabs per flock). Each three swabs collected from each flock were pooled in one tube and submitted to Veterinary Diagnostic Laboratory (Tehran, Iran).

**RNA extraction** Viral RNA was extracted from the directly pooled tracheal swabs in RLT buffer (Qiagen, Hilden, Germany) and 10 μL 2-mercaptoethanol (Merck, Darmstadt, Germany) per 1 mL buffer using RNeasy Mini Kit (Qiagen), according to the manufacturer’s protocol.

**Reverse transcription** The reverse transcriptation (RT) reaction was performed using ReverAid™ first strand cDNA synthesis kit (Thermo Scientific, Burlington, Canada), according to the product manual. The resultant cDNA was immediately used in a PCR or stored at –20 ˚C for later use.

**Amplification of the spike gene** Nested reverse transcription polymerase chain reaction (RT-PCR) was performed using spike gene primers as described previously to amplify 392 bp fragment of the spike gene.^30^ The first round of amplification (495 bp) was performed using SX1 (5ʹ-CACCTAGAGGTTTGT/CTA/TGCAT-´3) and SX2 (5ʹ-TCCACCTCTATAAACACCC/TTT-´3) primers. The PCR reaction was performed in 25 μL reaction mixture containing 1 μL dNTP (10 mM), 0.50 μL of each primer (25 pmol μL^-1^), 1 μL MgCl_2_ (50 mM), 2.50 μL 10X PCR buffer, 0.20 μL Taq DNA Polymerase, 2.50 μL cDNA and 16.80 μL dH_2_O (all from SinaClon, Tehran, Iran). The amplification was performed using 35 thermal cycles including 94 ˚C for 30 sec, 58 ˚C for 30 sec, and 72 ˚C for 30 sec. The PCR product was used as template for the second round of amplification in which SX3 (5´-TAATACTGGC/TAATTT TTCAGA-´3), and SX4 (5ʹ´AATACAGATTGCT TACAACCACC-´3) primers were used. The PCR reaction was carried out under the above condition.

**Agarose gel electrophoresis.** The PCR products were electrophoresed on 2% agarose gel and visualized by staining with 0.50 μg mL^-1^ ethidium bromide by UV transilluminator (M-15; UVP, Upland, USA).

**PCR product purification.** The PCR products were purified using PCR purification Kit (Roche, Mannheim, Germany) according to kit’s manufacture instructions.

**Nucleotide sequencing, deduced amino acid analysis and phylogenetic tree. **Purified RT-PCR products were sequenced by ABI Prism BigDye terminator cycle sequencing ready reaction kit (Applied Biosystems, Foster City, USA) in a forward direction using primer SX3 and in a reverse direction using primer RX4. Nucleotide sequence of the PCR product (392 bp), which was submitted to NCBI, were compared with the IBV sequences in GenBank database and sequence similarities were analyzed by BLAST. Multiple sequence alignments were carried out with Clustal W and phylogenetic tree was constructed with MEGA software (version 5; Biodesign Institute, Tempe, USA) using the Neighbor-joining tree method with 1000 bootstrap.^31^

**GenBank accession number of IBV sequence.** The partial S1 gene sequences of IBVs were submitted to the GenBank database under accession numbers KX578825-KX578834.

## Results

**Fig. 1:**
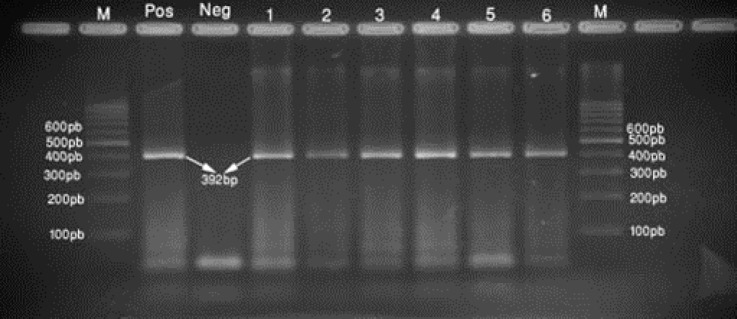
Agarose gel electrophoresis of the 392 bp RT-PCR product of the selected 6 isolates: lane 1, 2, 3, 4, 5, 6: the selected samples. M: represents the 100 bp molecular weight marker. Pos: positive control; Neg: negative control

The RNA was extracted and cDNA was synthesized, and further a (392bp) segment of the S1 gene was amplified by nested RT-PCR (Fig. 1). Samples from twelve farms (80%) found to be positive (Table 1).

**Table 1:** The history and areas of the samples collected for genotyping of infectious bronchitis viruses from broiler farms in Iran between 2014 - 2015

**Flock No.**	**Isolates ID**	**Area**	**Vaccination program**	**Collection date**	**Accession No.**
**1**	Iran/Bu/Mass/SH1229.2/15	Genaveh	H120 (Drink)	09.2015	KX578825
**2**	Iran/Bu/Variant2/SH1229.5/15	Bushehr	No	06.2015	KX578826
**3**	Iran/Bu/Variant2/SH1229.7/14	Bushehr	No	05.2014	KX578827
**4**	Iran/Bu/Variant2/SH1229.8/14	Bushehr	No	08.2014	KX578828
**5**	Iran/Bu/Variant2/SH1229.9/14	Burazjan	No	11.2014	KX578829
**6**	Iran/Bu/Mass/SH1229.13/14	Bushehr	H120 (Drop)	01.2014	KX578830
**7**	Iran/Bu/Variant2/SH1229.19/14	Genaveh	No	10.2014	KX578831
**8**	Iran/Bu/Variant2/SH1450.5/15	Bushehr	H120 (Drink)	02.2015	KX578832
**9**	Iran/Bu/Mass/SH1450.12/15	Bushehr	H120 (Drink)	04.2014	KX578833
**10**	Iran/Bu/Variant2/SH1450.19/15	Bushehr	H120 (Drop)	12.2015	KX578834
**11**	Negative	Bushehr	H120 (Drop)	-	-
**12**	Negative	Burazjan	H120 (Drink)	-	-
**13**	Negative	Bushehr	H120 (Drop)	-	-
**14**	Mix	Burazjan	No	-	-
**15**	Mix	Burazjan	No	-	-

**Phylogenetic Analysis. **To evaluate sequences variation among the positive samples, the sequences were compared with each other and with the reference sequences (Table 2). The results indicated a close relationship with Massachusetts and variant 2. The viruses from seven farms (46.6%) were identified as field viruses which were closely related to variant 2. The viruses from three farms (20.0%) were characterized as Mass type and related to vaccine strains. Two different IB viruses (variant 2 and Mass) were detected in samples from two farms (13.3%), simultaneously. The detected variant 2 genotype in Bushehr demonstrated a high similarity to variant 2 as those reported from the Middle East.

**Fig. 2:**
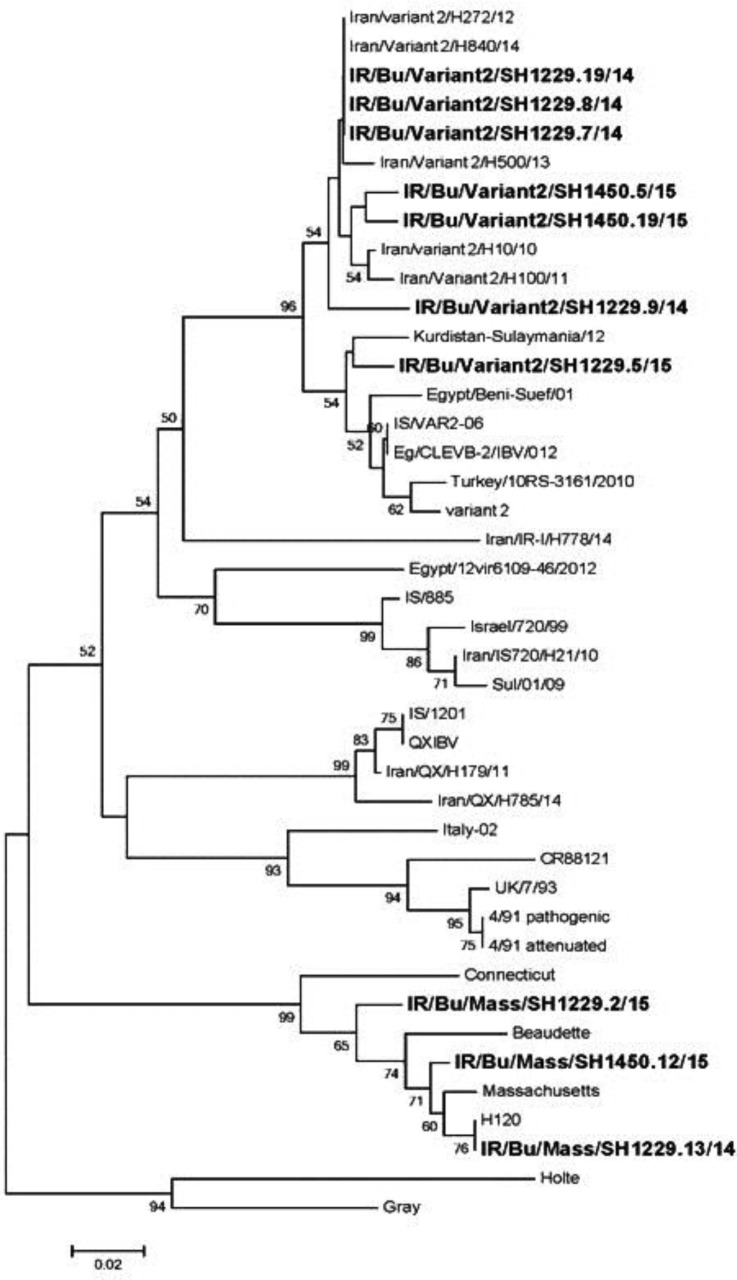
The phylogenic tree for IBV strains detected in the current study and other related isolates in the gene bank Middle East sequences using Mega-5 program. Analyses were based on S1 gene 392bp nucleotides

On the base gene sequences, phylogenetic tree was constructed from the nucleotide sequences of the S1 glycoprotein gene, revealing that the sequences of the recent Iranian strains formed two main groups (Fig. 2).

The first group was subdivided into two subgroups: one including Kurdistan-Sulaymania/12, Egypt/Beni Suef/ 01, Turkey/10RS-3161/2010, IS/VAR2-06, variant_2, Eg/ CLEVB-2/IBV/012, IR/Bu/variant2/SH1229.5/15 and the other one including IR/Bu/variant2/SH1450.5/15, IR/Bu/variant2/SH1450.19/15, IR/Bu/variant2/SH1229.7/14, IR/Bu/variant2/SH1229.8/14, IR/Bu/variant2/SH1229.9/14, IR/Bu/variant2/SH1229.19/14, Iran/variant2/H272/ 12, Iran/variant2/H840/14, Iran/variant2/H500/13, Iran/ variant2/H10/10, Iran/variant2/H100/11. 

The above findings indicated a high similarity to the Middle East variant 2.

These seven IBV field isolates showed 91.80% to 97.60% nucleotide sequence identity to IBV- strains Kurdistan-Sulaymania/12, Egypt/Beni-Suef/01, Turkey/ 10RS-3161/2010, IS/VAR2-06, variant 2, Eg/CLEVB-2/IBV/012 and Libya-06-2012. (Variant 2 like strain). Nucleotide sequence identity between second subgroup isolates ranged from 92.90% to 100% (Table 2).

Comparing mentioned isolates with vaccinal strains used in Iran (H120, Massachusetts and 4/91), the results showed 72.90% to 76.50%, 71.80% to 75.30% and 78.80% to 80% similarity (Table 2), respectively.

The second groups include IR/Bu/Mass/SH1229.2/15, IR/Bu/Mass/SH1229.13/14, IR/Bu/Mass/SH1450.12/15.

**Table 2:** Nucleotide identity percentage of ten selected IBV isolates in this study and other reference IBV strains from Gene bank.

**IBV Strain/Isolate**		**1**	**2**	**3**	**4**	**5**	**6**	**7**	**8**	**9**	**10**	**11**	**12**	**13**	**14**
**IS/VAR2-06**	**1**	100													
**Egypt/Beni-Suef/01**	**2**	97.60	100												
**Kurdistan-Sulaymania/12**	**3**	96.50	94.10	100											
**Turkey/10RS-3161/2010**	**4**	98.80	96.50	95.30	100										
**Variant2**	**5**	98.80	96.50	95.30	97.60	100									
**Eg/CLEVB-2/IBV/012**	**6**	100	97.60	96.50	98.80	98.80	100								
**Libya-06-2012**	**7**	100	97.60	96.50	98.80	98.80	100	100							
**IR/Bu/Variant2/SH1450.5/15**	**8**	92.90	91.80	94.10	91.80	91.80	92.90	92.90	100						
**IR/Bu/Variant2/SH1450.19/15**	**9**	94.10	92.90	95.30	92.90	92.90	94.10	94.10	97.60	100					
**IR/Bu/Variant2/SH1229.5/15**	**10**	97.60	95.30	96.50	96.50	96.50	97.60	97.60	92.90	94.10	100				
**IR/Bu/Variant2/SH1229.7/14**	**11**	95.30	94.10	94.10	94.10	94.10	95.30	95.30	97.60	97.60	95.30	100			
**IR/Bu/Variant2/SH1229.8/14**	**12**	95.30	94.10	94.10	94.10	94.10	95.30	95.30	97.60	97.60	95.30	100	100		
**IR/Bu/Variant2/SH1229.9/14**	**13**	92.90	91.80	91.80	91.80	91.80	92.90	92.90	94.10	94.10	92.90	96.50	96.50	100	
**IR/Bu/Variant2/SH1229.19/14**	**14**	95.30	94.10	94.10	94.10	94.10	95.30	95.30	97.60	97.60	95.30	100	100	96.50	100

## Discussion

It is imperative to recognize the prevalent strain(s) of infectious bronchitis virus in a region or country, and to select the best vaccine strain and the vaccination program for controlling the disease. The major problem in the immunization against IBV is the presence of various IBV serotypes in the field against available vaccines which cannot induce proper immunity. The aim of the present study was to detect and identify the type of prevailing IBV strains in Bushehr province. Previous studies comparing conventional and nested RT-PCR methods indicated that nested RT-PCR was more sensitive for detection of IBV.^32^^-^^34^ The implementation of nationwide genotyping of IBV strains is necessary to determine the distribution of virus genotypes and to develop and adopt suitable vaccination strategies. Antigenic characterization of IBV isolates is important for selecting new and appropriate vaccines for the corresponding geographical regions.^35^ New serotypes or variant strains may emerge due to only a few changes in the amino acid sequence of the S1protein.^3^ Therefore, the S1 gene of the isolates should be determined to differentiate field and vaccine isolates.^36^ Regarding the results obtained, 12 IBV isolates were identified. Phylogenetic analysis based on S1gene nucleotide sequences showed that most of the Iranian isolates belonged to two distinct groups.

Based on nucleotide sequencing of the S1 gene, a number of field isolates in the present study showed maximum similarity to Variant 2 (IS/1494/06 like strain). This is the first report of IBV variant 2 in the broiler flocks of Bushehr province. These variants displayed homologies ranging from 72.90% to 76.50%, and 78.80% to 80% with H120 and 4/91, respectively (Tab e 2).

The second group included three strains which were closely related to Massachusetts (Mass) type strains. In the present study, IR/variant2 viruses (IS/1494/06 like) were recognized as major dominant genotypes and the most important IBV type in Bushehr province chicken flocks. They shared the highest identity of 91.80 to 97.60% with Kurdistan-Sulaymania/12, Egypt/Beni-Suef/01, Turkey/10RS-3161/2010, IS/VAR2-06, variant 2, Eg/CLEVB-2/IBV/012 and Libya-06-2012. These findings are in agreement with those of Hosseini *et al*. who reported a genotype variant 2 (IS/1494/06 like) had been circulating in Iran between 2010 to 2014.^27^

Homayounimehr *et al.* reported IR/7/2011, R/8/2011, and IR/9/2011 isolates which appeared different from the mentioned IBV types and formed separate branches in the phylogenetic tree (variant 2).^29^ Mahmood *et al*. reported a new IBV isolate in the Kurdistan region from 2008 to 2010 that caused kidney lesions. Their results indicated the circulation of 793/B with variant 2 in poultry flocks in Iraq, which were similar to the findings obtained in the present study.^37^

Najafi *et al*. reported Variant 2-like viruses (IS/1494/06 like) that were the most predominant IBV type in Iranian chicken flocks. They shared the highest identity of 99.22% with IS/1494/06, Turkey/TR8, and Eg/ CLEVB-2/IBV/012. These findings had high similarity with our results.^28^

Our findings are also in agreement with several other studies carried out in the Middle East countries between 2004 and 2015. Some Iraqi researchers studied circulating viruses in Broiler farm and showed that these strains belong to variant 2 (IS/1494-lik) that had high nucleotide sequence identity with IBV isolates from Iran, Israel, Egypt, Turkey, and Kurdistan.^38^ The IB viruses in Egypt, Jordan, Turkey and Libya showed a close relationship to Israeli variants.^28^^,^^39^^-^^43^ Following the first report by Meir *et al*.,^44^ the variant 2 has been reported from some Middle East countries such as Iran.^27^^-^^29^^,^^42^^,^^43^ Since these countries have close connections (e.g. through language, religion, relation-ship, holy places, sectarian war, economic exchange, immigration etc.), so these connections can play an important role in spreading of this variant.

The first isolation of IBV in Iranian chicken flocks was reported in 1994.^25^ The present study is the first report on var2 IS/1494/06 in Bushehr province, in Iran, confirming the presence of the Var2 genotype. Ma5, H120, and attenuated 4/91 IBV-based vaccination strategies have been applied to IB control on poultry farms in Iran recently,^45^^,^^46^ and despite their use, diagnosis of IB in the vaccinated chickens is common. The results of this study may partially explain the failure of Massachusetts-type vaccines and therefore necessitates revising the Iranian vaccination strategy against infectious bronchitis. The low identity between most of Iranian isolates with Mass-type vaccine strain, the presence of variant 2, and other new genotypes 4/91 can be regarded as the causes of vaccination failure. 

Moreover, secondary infections and immuno-suppressive agents like infectious bursal disease virus and Chicken anemia virus may also lead to vaccination failure and consequently IBV outbreaks among poultry flocks. These substantial reasons can result in immune failure, poor cross-protection between the field virus and vaccine strain, and the continual emergence of new variants.^28^ Genotypes found in Sulaymania-Kurdistan, Iraq, included group A (very similar to Iranian isolates), and group C (similar to IS/1494 and Egypt/Beni-Seuf/01 isolates).^47^ These findings are in agreement with the present study.

Cross-protection between IBV strains depends on the amino acid similarity of S1. Based on S1 glycoprotein amino acid sequence, Iranian IBV’s homology with H120 vaccine, Massachusetts vaccine and 793/B vaccine ranges from 72.90% to 76.50%, from 71.80% to 75.30% and from 78.80% to 80.00%, respectively. These findings explain the poor vaccine performance in the field and show that the disease outbreaks were associated with IBV variants, which circumvent vaccination immunity. Further, the findings emphasize the need for new control strategies of IBV in Iran.

In summary, the present study is the first report of IBV in Bushehr, Iran, illuminating the circulation of a variant of IBV genotype in chicken farms. Heterogeneity with vaccine strains can explain a poor vaccination performance and disease outbreak in this area. The results emphasize the need for new control strategies and re-arrangement of preventative measure of IBV in Bushehr, Iran.
